# Hexaaqua­cobalt(II) 2,2′-[naphthalene-1,8-diylbis(­oxy)]diacetate dihydrate

**DOI:** 10.1107/S1600536813000512

**Published:** 2013-01-12

**Authors:** Hui Fang Shi, Tao Wu, Peng Gang Jiang, Zhi Hao, Miao Miao Zhang

**Affiliations:** a Department of Chemistry, College of Chemistry and Chemical Engineering, Chongqing University, Chongqing 400030, People’s Republic of China

## Abstract

In the title compound, [Co(H_2_O)_6_](C_14_H_10_O_6_)·2H_2_O, the 2,2′-[naphthalene-1,8-diylbis(­oxy)]diacetate dianion *L* is not coordinated to the Co^II^ ion. The asymmetric unit contains half of the *L* dianion, half of a [Co(H_2_O)_6_]^2+^ cation (both molecules being completed by inversion symmetry), and one water mol­ecule. The crystal packing features O—H⋯O hydrogen bonding between the carboxyl­ate groups, the aqua ligands and the hydrate water mol­ecules.

## Related literature

In recent years, metal complexes have been synthezised with potential applications in mol­ecular sorption, electrical conductivity, catalysis, magnetism, non-linear optics and mol­ecular sensing, see: James (2003 ▶); Murray *et al.* (2009 ▶); Karmakar *et al.* (2009 ▶); Kurmoo (2009 ▶); Bradshaw *et al.* (2005 ▶). The 5-carboxymethoxy-naphtalene1-yl(oxy)-acetate ligand can provide a dominant packing feature and it often controls the supra­molecular assembly, see: Desiraju (2007 ▶). For Cd complexes with different co-ligands, see: Deka *et al.* (2011 ▶); Li *et al.* (2012 ▶) and for Zn complexes, see: Mondal *et al.* (2008 ▶).
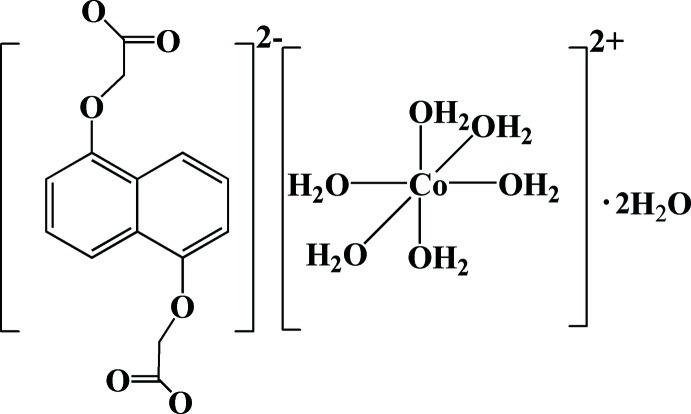


## Experimental

### 

#### Crystal data

[Co(H_2_O)_6_](C_14_H_10_O_6_)·2H_2_O
*M* *_r_* = 477.28
Triclinic, 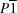

*a* = 6.377 (2) Å
*b* = 6.642 (2) Å
*c* = 12.979 (5) Å
α = 79.669 (10)°
β = 79.963 (11)°
γ = 64.911 (8)°
*V* = 486.8 (3) Å^3^
*Z* = 1
Mo *K*α radiation
μ = 0.95 mm^−1^
*T* = 293 K
0.30 × 0.28 × 0.25 mm

#### Data collection

Siemens CCD area-detector diffractometer
Absorption correction: multi-scan (*SADABS*; Sheldrick, 2007 ▶) *T*_min_ = 0.731, *T*_max_ = 1.000
3126 measured reflections
1678 independent reflections
1605 reflections with *I* > 2σ(*I*)
*R*_int_ = 0.018

#### Refinement

*R*[*F* ^2^ > 2σ(*F* ^2^)] = 0.031
*wR*(*F* ^2^) = 0.080
*S* = 1.09
1678 reflections
161 parameters
12 restraints
H atoms treated by a mixture of independent and constrained refinement
Δρ_max_ = 0.37 e Å^−3^
Δρ_min_ = −0.48 e Å^−3^

### 

Data collection: *SMART* (Siemens, 1996 ▶); cell refinement: *SAINT* (Siemens, 1996 ▶); data reduction: *SAINT*; program(s) used to solve structure: *SHELXS97* (Sheldrick, 2008 ▶); program(s) used to refine structure: *SHELXL97* (Sheldrick, 2008 ▶); molecular graphics: *SHELXTL* (Sheldrick, 2008 ▶); software used to prepare material for publication: *SHELXTL*.

## Supplementary Material

Crystal structure: contains datablock(s) I, global. DOI: 10.1107/S1600536813000512/vm2185sup1.cif

Structure factors: contains datablock(s) I. DOI: 10.1107/S1600536813000512/vm2185Isup2.hkl

Additional supplementary materials:  crystallographic information; 3D view; checkCIF report

## Figures and Tables

**Table 1:** Selected bond lengths (Å)

Co1—O4	2.056 (2)
Co1—O5	2.0792 (17)
Co1—O6	2.093 (2)

**Table 2:** Hydrogen-bond geometry (Å, °)

*D*—H⋯*A*	*D*—H	H⋯*A*	*D*⋯*A*	*D*—H⋯*A*
O6—H6*C*⋯O7^i^	0.96 (2)	1.76 (3)	2.723 (3)	174 (3)
O6—H6*D*⋯O7^ii^	0.93 (2)	1.83 (3)	2.751 (3)	171 (3)
O5—H5*A*⋯O2^iii^	0.93 (3)	1.96 (3)	2.850 (3)	159 (2)
O5—H5*B*⋯O3^iv^	0.94 (3)	1.87 (2)	2.783 (3)	165 (2)
O7—H7*A*⋯O3^v^	0.92 (2)	1.82 (3)	2.736 (3)	171 (3)
O7—H7*B*⋯O2^vi^	0.93 (3)	1.89 (3)	2.780 (3)	158 (2)

Symmetry codes: (i) 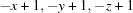; (ii) 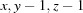; (iii) 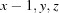; (iv) 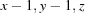; (v) 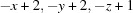; (vi) 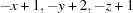.

**Fig. 1.**
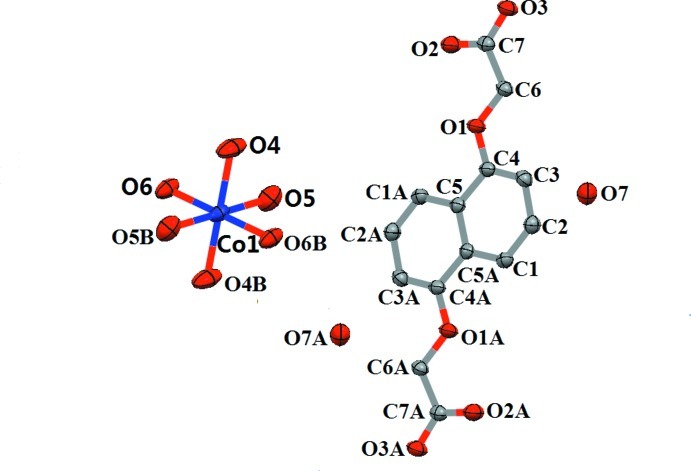
The molecular structure of (I), with the atomic numbering scheme and displacement ellipsoids at the 50% probability level (H atoms omitted for clarity) [symmetry codes: (A) -x + 1, -y + 1, -z + 1, (B) -x, -y + 1, -z.].

**Fig. 2.**
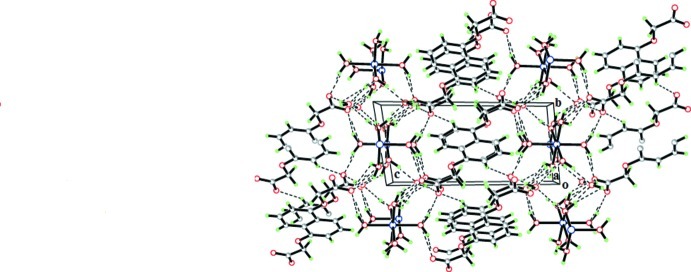
Three dimensional supramolecular architecture constructed by intermolecular hydrogen bonds. The dotted lines indicate the hydrogen bonds.

## supplementary crystallographic information

### Comment

In recent years, metal complexes have been synthezised with potential applications in molecular sorption, electrical conductivity, catalysis, magnetism, nonlinear optics, and molecular sensing (James, 2003; Murray *et al.*, 2009; Kurmoo, 2009; Karmakar *et al.*, 2009; Bradshaw *et al.*, 2005). The *LH2* ligand (5-carboxymethoxy-naphtalen-1-yloxy)-acetic acid) has received our attention because it can provide a dominant packing feature and it often controls the supramolecular assembly (Desiraju *et al.*, 2007). At present, many of its metal complexes have already been reported, but most are focused on Cd complexes (Deka *et al.*, 2011; Li *et al.*, 2012) and Zn complexes (Li *et al.*, 2012) with different co-ligands such as 2,2-bipyridine or 1,10-phenanthroline (phen). In the present paper, we hydrothermally synthesized a novel coordination complex constructed by Co^II^, *L* and water molecules and determined its crystal structure (Fig. 1).

The asymmetric unit of the complex consists of a half ligand *L*, a half Co^II^ ion complexed with three water molecules and one additional water molecule. The Co^II^ center is octahedrally coordinated by six water molecules. The two carboxylate arms of the *LH2* ligand lie in the same plane as the naphthalene ring. The hydrogen atoms of the water molecular and the oxygen atoms which are coordinated by Co^II^ are involved in hydrogen bonding with the oxygen atoms of the carboxylate group (Table 2, Fig. 2). In this case a sheet-like structure is formed.

### Experimental

The ligand LH2 was synthesized according to the procedure published by Mondal *et al.* (2008).

A mixture of Co(NO_3_)_2_.6H_2_O (0.05 mmol, 0.015 g), *L* (0.05 mmol, 0.013 g), water (1 ml) and DMF (1 ml) was heated at 393 K in a Teflon-lined autoclave for three days, followed by slow cooling to room temperature. The resulting pink block crystals were filtered off and washed with distilled water.

### Refinement

The H atoms on the ligands were positioned geometrically and refined as riding [C–H = 0.93 Å and *U*_iso_(H) = 1.2*U*_eq_(C)]. Hydrogen atoms of the water molecules were located in the Fourier difference maps and refined with restraints for the O–H distances and H–O–H angles.

### Crystal data

**Table:**

[Co(H_2_O)_6_](C_14_H_10_O_6_)·2H_2_O	*Z* = 1
*M**_r_* = 477.28	*F*(000) = 249
Triclinic, *P*1	*D*_x_ = 1.628 Mg m^−^^3^
Hall symbol: -P 1	Mo *K*α radiation, λ = 0.71073 Å
*a* = 6.377 (2) Å	Cell parameters from 1414 reflections
*b* = 6.642 (2) Å	θ = 3.2–27.5°
*c* = 12.979 (5) Å	µ = 0.95 mm^−^^1^
α = 79.669 (10)°	*T* = 293 K
β = 79.963 (11)°	Block, pink
γ = 64.911 (8)°	0.30 × 0.28 × 0.25 mm
*V* = 486.8 (3) Å^3^	

### Data collection

**Table:**

Siemens CCD area-detector diffractometer	1678 independent reflections
Radiation source: fine-focus sealed tube	1605 reflections with *I* > 2σ(*I*)
Graphite monochromator	*R*_int_ = 0.018
φ and ω scans	θ_max_ = 25.0°, θ_min_ = 3.4°
Absorption correction: multi-scan (*SADABS*; Sheldrick, 2007)	*h* = −7→7
*T*_min_ = 0.731, *T*_max_ = 1.000	*k* = −7→7
3126 measured reflections	*l* = −15→15

### Refinement

**Table:**

Refinement on *F*^2^	Primary atom site location: structure-invariant direct methods
Least-squares matrix: full	Secondary atom site location: difference Fourier map
*R*[*F*^2^ > 2σ(*F*^2^)] = 0.031	Hydrogen site location: inferred from neighbouring sites
*wR*(*F*^2^) = 0.080	H atoms treated by a mixture of independent and constrained refinement
*S* = 1.09	*w* = 1/[σ^2^(*F*_o_^2^) + (0.0408*P*)^2^ + 0.1833*P*] where *P* = (*F*_o_^2^ + 2*F*_c_^2^)/3
1678 reflections	(Δ/σ)_max_ < 0.001
161 parameters	Δρ_max_ = 0.37 e Å^−^^3^
12 restraints	Δρ_min_ = −0.48 e Å^−^^3^

### Special details

**Table:**

Geometry. All e.s.d.'s (except the e.s.d. in the dihedral angle between two l.s. planes) are estimated using the full covariance matrix. The cell e.s.d.'s are taken into account individually in the estimation of e.s.d.'s in distances, angles and torsion angles; correlations between e.s.d.'s in cell parameters are only used when they are defined by crystal symmetry. An approximate (isotropic) treatment of cell e.s.d.'s is used for estimating e.s.d.'s involving l.s. planes.
Refinement. Refinement of *F*^2^ against ALL reflections. The weighted *R*-factor *wR* and goodness of fit *S* are based on *F*^2^, conventional *R*-factors *R* are based on *F*, with *F* set to zero for negative *F*^2^. The threshold expression of *F*^2^ > σ(*F*^2^) is used only for calculating *R*-factors(gt) *etc*. and is not relevant to the choice of reflections for refinement. *R*-factors based on *F*^2^ are statistically about twice as large as those based on *F*, and *R*- factors based on ALL data will be even larger.

### Fractional atomic coordinates and isotropic or equivalent isotropic displacement parameters (Å^2^)

**Table:**

	*x*	*y*	*z*	*U*_iso_*/*U*_eq_	
O1	0.7658 (2)	0.7636 (3)	0.38143 (11)	0.0299 (3)	
O2	0.9252 (3)	0.9376 (3)	0.20485 (12)	0.0374 (4)	
O3	1.0787 (3)	1.1022 (3)	0.28675 (12)	0.0331 (4)	
C5	0.5504 (3)	0.5494 (3)	0.45495 (15)	0.0222 (4)	
C4	0.6759 (3)	0.6729 (3)	0.47211 (15)	0.0239 (4)	
C3	0.6996 (3)	0.6953 (3)	0.57172 (16)	0.0275 (4)	
H3	0.7826	0.7752	0.5816	0.033*	
C2	0.5966 (3)	0.5961 (4)	0.65934 (16)	0.0282 (4)	
H2	0.6115	0.6131	0.7269	0.034*	
C1	0.4761 (3)	0.4762 (3)	0.64724 (15)	0.0257 (4)	
H1	0.4113	0.4111	0.7062	0.031*	
C6	0.8919 (3)	0.8899 (3)	0.39326 (15)	0.0254 (4)	
H6A	1.0262	0.7948	0.4304	0.031*	
H6B	0.7933	1.0118	0.4343	0.031*	
C7	0.9709 (3)	0.9827 (3)	0.28607 (16)	0.0255 (4)	
Co1	0.0000	0.5000	0.0000	0.03054 (16)	
O7	0.5069 (3)	1.0109 (3)	0.83972 (12)	0.0387 (4)	
O5	0.0045 (3)	0.5054 (3)	0.15926 (12)	0.0407 (4)	
O6	0.3582 (3)	0.3007 (3)	−0.00989 (14)	0.0504 (5)	
H6C	0.403 (6)	0.199 (5)	0.0532 (16)	0.075 (10)*	
H6D	0.411 (6)	0.214 (5)	−0.0654 (18)	0.087 (12)*	
H5A	−0.021 (6)	0.630 (4)	0.191 (2)	0.076 (10)*	
H5B	0.013 (5)	0.385 (4)	0.211 (2)	0.067 (9)*	
H7A	0.638 (3)	0.982 (5)	0.7915 (19)	0.062 (9)*	
H7B	0.380 (4)	1.029 (6)	0.807 (2)	0.085 (11)*	
O4	0.0880 (5)	0.7707 (3)	−0.03180 (15)	0.0629 (6)	
H4A	0.2060	0.7419	−0.0738	0.094*	
H4B	−0.043 (5)	0.897 (9)	−0.061 (4)	0.27 (4)*	

### Atomic displacement parameters (Å^2^)

**Table:**

	*U*^11^	*U*^22^	*U*^33^	*U*^12^	*U*^13^	*U*^23^
O1	0.0387 (8)	0.0372 (9)	0.0246 (7)	−0.0275 (7)	−0.0017 (6)	−0.0010 (6)
O2	0.0532 (9)	0.0444 (10)	0.0277 (8)	−0.0338 (8)	−0.0041 (7)	−0.0010 (7)
O3	0.0386 (8)	0.0346 (9)	0.0351 (8)	−0.0253 (7)	−0.0006 (6)	−0.0033 (6)
C5	0.0206 (8)	0.0211 (10)	0.0238 (9)	−0.0079 (7)	−0.0025 (7)	−0.0012 (7)
C4	0.0240 (9)	0.0241 (10)	0.0250 (10)	−0.0128 (8)	−0.0005 (7)	−0.0005 (8)
C3	0.0293 (10)	0.0295 (11)	0.0299 (11)	−0.0170 (8)	−0.0041 (8)	−0.0044 (8)
C2	0.0337 (10)	0.0323 (11)	0.0223 (10)	−0.0157 (9)	−0.0039 (8)	−0.0049 (8)
C1	0.0270 (9)	0.0288 (11)	0.0229 (10)	−0.0143 (8)	−0.0006 (7)	−0.0014 (8)
C6	0.0285 (9)	0.0254 (10)	0.0274 (10)	−0.0160 (8)	−0.0027 (8)	−0.0027 (8)
C7	0.0260 (9)	0.0231 (10)	0.0284 (11)	−0.0117 (8)	−0.0013 (8)	−0.0023 (8)
Co1	0.0457 (3)	0.0224 (2)	0.0229 (2)	−0.01429 (18)	−0.00138 (17)	−0.00230 (16)
O7	0.0332 (8)	0.0466 (10)	0.0329 (9)	−0.0127 (7)	−0.0026 (7)	−0.0068 (7)
O5	0.0655 (11)	0.0304 (9)	0.0257 (8)	−0.0194 (8)	−0.0046 (7)	−0.0026 (7)
O6	0.0605 (11)	0.0451 (11)	0.0359 (10)	−0.0118 (9)	−0.0026 (8)	−0.0085 (8)
O4	0.1209 (18)	0.0469 (12)	0.0407 (10)	−0.0550 (13)	−0.0101 (11)	0.0015 (9)

### Geometric parameters (Å, º)

**Table:**

O1—C4	1.371 (2)	C6—H6B	0.9700
O1—C6	1.427 (2)	Co1—O4	2.056 (2)
O2—C7	1.257 (3)	Co1—O4^ii^	2.056 (2)
O3—C7	1.253 (3)	Co1—O5^ii^	2.0792 (17)
C5—C1^i^	1.414 (3)	Co1—O5	2.0792 (17)
C5—C5^i^	1.425 (4)	Co1—O6	2.093 (2)
C5—C4	1.431 (3)	Co1—O6^ii^	2.093 (2)
C4—C3	1.368 (3)	O7—H7A	0.921 (17)
C3—C2	1.413 (3)	O7—H7B	0.932 (17)
C3—H3	0.9300	O5—H5A	0.931 (17)
C2—C1	1.362 (3)	O5—H5B	0.933 (17)
C2—H2	0.9300	O6—H6C	0.962 (17)
C1—C5^i^	1.414 (3)	O6—H6D	0.929 (18)
C1—H1	0.9300	O4—H4A	0.8200
C6—C7	1.510 (3)	O4—H4B	0.97 (2)
C6—H6A	0.9700		
			
C4—O1—C6	116.91 (15)	O4—Co1—O4^ii^	180.0
C1^i^—C5—C5^i^	119.8 (2)	O4—Co1—O5^ii^	91.63 (7)
C1^i^—C5—C4	122.26 (18)	O4^ii^—Co1—O5^ii^	88.37 (7)
C5^i^—C5—C4	117.9 (2)	O4—Co1—O5	88.37 (7)
C3—C4—O1	124.53 (18)	O4^ii^—Co1—O5	91.63 (7)
C3—C4—C5	121.27 (18)	O5^ii^—Co1—O5	180.00 (11)
O1—C4—C5	114.19 (17)	O4—Co1—O6	86.53 (10)
C4—C3—C2	119.38 (19)	O4^ii^—Co1—O6	93.47 (10)
C4—C3—H3	120.3	O5^ii^—Co1—O6	91.34 (7)
C2—C3—H3	120.3	O5—Co1—O6	88.66 (7)
C1—C2—C3	121.62 (18)	O4—Co1—O6^ii^	93.47 (10)
C1—C2—H2	119.2	O4^ii^—Co1—O6^ii^	86.53 (10)
C3—C2—H2	119.2	O5^ii^—Co1—O6^ii^	88.66 (7)
C2—C1—C5^i^	119.98 (18)	O5—Co1—O6^ii^	91.34 (7)
C2—C1—H1	120.0	O6—Co1—O6^ii^	180.00 (7)
C5^i^—C1—H1	120.0	H7A—O7—H7B	110 (2)
O1—C6—C7	109.63 (16)	Co1—O5—H5A	126.3 (19)
O1—C6—H6A	109.7	Co1—O5—H5B	123.8 (18)
C7—C6—H6A	109.7	H5A—O5—H5B	109 (2)
O1—C6—H6B	109.7	Co1—O6—H6C	112.4 (19)
C7—C6—H6B	109.7	Co1—O6—H6D	113 (2)
H6A—C6—H6B	108.2	H6C—O6—H6D	107 (2)
O3—C7—O2	125.29 (19)	Co1—O4—H4A	109.5
O3—C7—C6	115.32 (17)	Co1—O4—H4B	107 (4)
O2—C7—C6	119.39 (18)	H4A—O4—H4B	111.3

Symmetry codes: (i) −*x*+1, −*y*+1, −*z*+1; (ii) −*x*, −*y*+1, −*z*.

### Hydrogen-bond geometry (Å, º)

**Table:**

*D*—H···*A*	*D*—H	H···*A*	*D*···*A*	*D*—H···*A*
O6—H6*C*···O7^i^	0.96 (2)	1.76 (3)	2.723 (3)	174 (3)
O6—H6*D*···O7^iii^	0.93 (2)	1.83 (3)	2.751 (3)	171 (3)
O5—H5*A*···O2^iv^	0.93 (3)	1.96 (3)	2.850 (3)	159 (2)
O5—H5*B*···O3^v^	0.94 (3)	1.87 (2)	2.783 (3)	165 (2)
O7—H7*A*···O3^vi^	0.92 (2)	1.82 (3)	2.736 (3)	171 (3)
O7—H7*B*···O2^vii^	0.93 (3)	1.89 (3)	2.780 (3)	158 (2)

Symmetry codes: (i) −*x*+1, −*y*+1, −*z*+1; (iii) *x*, *y*−1, *z*−1; (iv) *x*−1, *y*, *z*; (v) *x*−1, *y*−1, *z*; (vi) −*x*+2, −*y*+2, −*z*+1; (vii) −*x*+1, −*y*+2, −*z*+1.
